# “We are really starving for respect and support,” the struggle of Iranian nurses in adhering to professional values: A qualitative study

**DOI:** 10.1002/nop2.1595

**Published:** 2023-01-30

**Authors:** Sahar Kazemi, Naser Parizad, Hossein Habibzadeh

**Affiliations:** ^1^ School of Nursing and Midwifery Urmia University of Medical Sciences Urmia Iran; ^2^ Patient Safety Research Center, Clinical Research Institute, Nursing & Midwifery School Urmia University of Medical Sciences Urmia Iran

**Keywords:** clinical, ethics, nurses, professional, qualitative study, values

## Abstract

**Aims:**

To investigate nurses' experiences of adhering to professional values in clinical settings.

**Design:**

A qualitative study with a conventional content analysis approach.

**Methods:**

This study was conducted from January 2021 to March 2022. Semi‐structured interviews were conducted with 12 nurses working in different wards of five public and private hospitals in West Azerbaijan of Iran. Data were analysed using the conventional content analysis approach proposed by Graneheim and Lundman (Nurse education today, 24, 2004, 105)

**Results:**

“Barriers to nurses' professional values” emerged as the main category of Iranian nurses' experiences in adhering to professional values. Three subcategories of barriers were revealed: “nurses' challenges,” “professional suppressors” and “poor working conditions.”

**Conclusion:**

Barriers in clinical settings can overshadow nurses' professional performance and disrupt their adherence to professional values. Nursing managers must pay attention to nurses' challenges, their professional suppressors and poor working condition to help them promote their professional performance in clinical settings. Thus, nursing managers should not neglect the continuous education of nurses to assist them in increasing their clinical skills by holding practical and theoretical workshops. Improving the working conditions and clinical atmosphere by recruiting a capable workforce and applying psychological and financial support for nurses are essential to increase the quality of nursing care.

## 1 INTRODUCTION

In this age of globalization, the development of health science and technology and the improvement of society's social and economic level have led to an increased need for excellent health services, including nursing care (Abdullah & Chong, 2019; Borzuoi & Mahnaz, 2018). The nursing profession is practiced worldwide in different cultures and environments, and adherence to professional ethics and values is an important goal in providing quality nursing care to the community (Sibandze & Scafide, 2018). Nurses are dynamic and committed people who are considered one of the most trusted professions in society. The level of trust attributed to this profession comes directly from the values that are expected to show in their practice (Costello, 2017).

The word value is considered as fundamental beliefs about what is right, good or desirable that motivates one's social and professional behaviour from an ethical perspective (Erkus & Dinc, 2018). Every professional group has basic standards called professional values (Lai & Lim, 2012). These values are considered a guide for professional behaviour for members of a particular profession and create a standard structure for attitudes and beliefs that affect behaviours (Lai & Lim, 2012; Poorchangizi et al., 2017). Also, as the largest professionals in the health care system, nurses have well‐known and important professional values (Dehghani et al., 2015), which the nursing profession is based on (Erkus & Dinc, 2018).

Since Florence Nightingale's time, professional ethics and values have been one of the basic elements of this profession and have been recognized as the basis of nursing care (Kangasniemi et al., 2015). Vocational values are acquired during socialization in nursing through ethical codes, nursing experiences, teachers and peers (Al‐Banna, 2017). According to Fagermoen (1997), the nursing profession's values, in addition to guiding nurses in decision‐making, form the professional identity of a nurse, which reflects their professional philosophy. Using these values in nursing practice increases the quality of patient care and job satisfaction, keeping nurses in the nursing profession and enhancing their commitment to the organization (Dehghani et al., 2015). In this regard, the study of Skela‐Savič et al. (2017) considered independence and professional competence as the most important professional values in creating knowledge and skills in evidence‐based practice and thus improve the quality of nursing care. In another study, nurses felt more valuable in their profession by using their scientific knowledge to improve patients' condition by adhering to professional values such as altruism (van der Wath & van Wyk, 2020). The nursing profession's values in Iran are similar to the nursing profession's values in other countries. However, limited financial and structural resources, cultural values and the general image of nursing may play a role in implementing these values (Dehghani et al., 2016). Identifying and managing nurses' challenges in practicing professional values in clinical settings based on their experiences is crucial. A qualitative study can assist in better understanding professional values as a complex concept with vast dimensions. Thus, this qualitative study with a content analysis approach was conducted for the first time in Iran to explore Iranian nurses' experiences of adhering to professional values in clinical settings and contributing to the relevant literature.

## 2 METHODS

### 2.1 Study design and research environment

This qualitative study was performed using the conventional content analysis approach (Hsiu‐Fang & Shannon, 2005) in hospitals and clinics of Urmia from January 2021 to March 2022.

### 2.2 Participants

The lead researcher recruited nurses who worked in medical and surgical wards, emergency departments and intensive care units of private and public hospitals in Urmia. Purposeful sampling was used to select participants, and the first three participants were selected from experienced nurses. Nurses with at least 1 year of clinical experience or those with experiences related to adherence to professional values in the clinical environment were considered experienced nurses in this study. In total, 12 participants consented to be interviewed Sampling continued until data saturation was met when no new information emerged from the three consecutive interviews (Saunders et al., 2018). Participants were selected from different genders, education levels, job positions and various cultural backgrounds to take a more comprehensive view of the phenomenon under study (Roulston, 2018).

### 2.3 Data collection

Data were collected using interviews and field notes, from March 2021 to December 2021. Before beginning the interview, the lead researcher arranged a short ten‐minute meeting or telephone call with the participants to establish an initial acquaintance and explain the purpose of the study. If they were interested in participating, the time and place of the interview were determined. The participants signed a written consent form to participate in the study and permitted their interviews to be recorded. All interviews were conducted in the privacy of the staff room where they work. The lead researcher conducted all face‐to‐face interviews individually. After establishing a friendly relationship and gaining the initial trust of the participants, the interviews began using a general question: “Please tell me about your experience in dealing with situations that have challenged your adherence to professional values?” and probing questions were asked such as “explain this further,” “Can you tell me more about that?” and “what do you mean by that?” according to their answers to obtain clarifications. In addition to the interviews, the second author took field notes to explore this concept more purposefully and asked more detailed questions (Hsiu‐Fang & Shannon, 2005). The average interview time was 45 min (30–60 min), and there was no need for repeated interviews with the participants in the study.

### 2.4 Trustworthiness

Guba and Lincoln's criteria were used to ensure the accuracy of the data (Lincoln & Guba, 1985). To confirm dependability, the entire research process was recorded and reported carefully. The credibility was ensured by prolonged interaction with participants, review of findings with experts (external audits) and participants (member checking), and complete immersion in the data. The transferability of the findings was also obtained by providing a detailed description of the research report and reviewing the findings by two experienced nurses who did not participate in the study. An audit trail was used, and an accurate recording of the study process and findings was performed to achieve confirmability and show the lack of researcher bias.

### 2.5 Data analysis

Recorded interviews were listened carefully several times and transcribed verbatim. The collected data were analysed through a conventional content analysis approach (Hsiu‐Fang & Shannon, 2005) proposed by Graneheim and Lundman (2004) (Graneheim & Lundman, 2004). According to this method, paragraphs, sentences and words were considered—units of meaning. A unit of meaning refers to a set of words and sentences that are conceptually related to each other. They are classified according to content and context. The written texts were reviewed several times to identify key concepts or units of meaning and extract the initial codes. The research team reviewed codes repeatedly, from code extraction to labeling. Similar codes were merged, categorized and labelled to obtain the subcategories. Finally, the extracted subcategories were compared and merged (if possible) to form the main category. The whole coding process was performed using MaxQda 10 software. The research team arranged several meetings and discussions about the issues before reaching an agreement on the coding and categorization. A total of 1902 codes were extracted from the analysis of the interviews, and one main category, three subcategories and 15 primary concepts were identified (Table 2). The Consolidated criteria for reporting qualitative studies (COREQ) checklist was used to enhance quality reporting in this research (see Appendix S1).

### 2.6 Ethical considerations

The protocol for the research was registered and approved by the ethical committee of Urmia University of Medical Sciences, Iran (IR.UMSU.REC.1399.331). All procedures in this study involving human participants were performed in accordance with the Declaration of Helsinki. All participants were fully informed about the purpose of the study, research method and right to leave the study at any time. Before the interview, the lead researcher provided the participants with information about anonymity and the confidentiality of the information. Then, written informed consent was obtained from all participants.

## 3 RESULTS

### 3.1 Demographic characteristics of participants

Five male nurses and seven female nurses were interviewed. Participants were four head nurses and eight clinical nurses (10 in public hospitals and two in private hospitals). Four participants had a master's degree, and eight had a bachelor's degree in nursing (Table 1).

**TABLE 1:** Demographic characteristics of participants

Variables	Mean	Maximum	Minimum
Age of participants	31.5±6.8	26	44
Seniority of work	10.42±6.35	4	20

### 3.2 Categories

Iranian nurses' experiences in adhering to professional values constitute the main category of “Barriers to nurses' professional values,” which was supported by the following subcategories: *nurses' challenges*, *professional suppressors* and *poor working conditions*. Nurses' challenges are supported by poor knowledge and skills of nurses, inexperience in nurses, challenges of novice nurses, routineness and dailiness in nursing practice, conflict of values and poor nurses' autonomy. Professional suppressors included physician dominance, suppression in the professional environment, violence against nurses and discrimination against nurses. Heavy workload, hostile work environment, lack of equipment and nursing shortage constituted poor working conditions (Table 2).

**TABLE 2:** Main category, subcategories and primary concepts

Main category	Subcategories	Primary concepts
Barriers to nurses' professional values	Nurses' challenges	Poor knowledge and skills of nurses Inexperience in nurses Challenges of novice nurses Routineness and dailiness in nursing practice Conflict of values Poor nurses' autonomy
	Clinical suppressors	Physician dominance Suppression in the professional environment Violence against nurses Discrimination against nurses Insufficient support for nurses
	Poor working conditions	Heavy workload Hostile work environment Lack of equipment Nursing shortage

#### 3.2.1 Nurses' challenges

Many participants believed that professional values may not be observed due to the nurses' lack of skill and knowledge. Some participants stated that their lack of experience led to poor nursing care, endangering patients' safety and making nurses unable to adhere to professional values. The lack of knowledge and skills was also reported among novice nurses, especially in the early years of their clinical practice when they experienced confusion, conflict and being harassed in the clinical setting, which made them unable to adhere to professional values and reluctant to attend the patient's bedside.

When I told my colleague in the hemodialysis ward, "Your patient was yawning.” He said, “What should I do with his yawning?” I told him that the patient might be sleepy as he is repeatedly yawning, and his blood pressure was more likely dropped and he must go and check his blood pressure. I do not think he has enough knowledge and skills in assessing his patients. (P 1)

My patient was bleeding from a jugular vein catheter, and I didn't even once check on my patient and see if everything was okay; at the end of hemodialysis, I saw blood all over the patient's bed, so I had to request for emergency packed red blood cells transfusion. (P 2)

I was in the ward as a new nurse. My friend called me and asked for personal information about one of the patients. I did not know that I should not give the patient's information to anyone. Unfortunately, I got into a lot of trouble. (P 7)

Routineness and dailiness in nursing practice were also reported as a challenge to them. Some participants talked about the experience of reduced accuracy and attention to proper patient care due to routineness and dailiness. Participants also reported that some older nurses experienced knowledge recession and lacked the motivation to improve themselves due to their day‐to‐day work. Some nurses are tired and suffered from dailiness, and they no longer care about the necessity of care.

Just yesterday, one of my high senior colleagues didn't change a patient's dressing despite knowing that he had to change his dressing and telling him that it was no problem, nothing would happen since the dressing was not opened. (p 10)

That's how I am; this is how I do things. Sometimes you need to bypass the rules and instructions. After working for these years, the work seniority goes forward and determines the right actions. (p 6)

Another challenge reported by the participants was a conflict between personal family and community values and their professional values that made them difficult to adhere to ethical behaviour and made decisions based on professional values.

One of my Muslim colleagues had a Christian patient last week. She reluctantly approached him and was very careful not to touch his body secretions because she believed they were unclean. (P11)

Our patient had died. The doctor asked me to disconnect the devices, but I did not want to touch the patient because touching the dead person's body would make me unclean in my religious beliefs. I really did not know what to do at that moment. (P2)

Having a lack of autonomy was a challenge for the participants. They believed that poor nurse autonomy is an important factor that causes lower self‐esteem in nurses compared with other health professionals.

I assessed the patient's condition and noticed that he was not stable enough to transfer to echocardiography, and he might experience cardio‐respiratory arrest on the way to echocardiography. I explained this issue to the head nurse and discussed that the patient should be transferred under close monitoring and CPR equipment. But she said: "nothing wrong will happen, don't exaggerate. (P3)

#### 3.2.2 Professional suppressors

A number of clinical suppressors were reported that impeded nurses' adherence to professional values. One outstanding factor was physicians' dominance and its negative impact on the work process and nurses' capabilities in clinical settings. Such a concern was seen as suppressing the development of decision‐making power in nurses.

One of the infants needed intubation during the previous night shift. We informed the anesthesiologist, and he intubated the patient. I listened to his lungs to make sure it was correctly placed and told the doctor that I did not hear any sound of air coming in and out of his lungs. But he said with confidence: “No, that's right! Just take care of your own business.” A few minutes later, the oxygen saturation level dropped, and the patient had a cardiac arrest. (p 12)

Another factor was related to nurses' experiencing suppression of their motivation for promotion and growth in the health care system and lack of support from their nursing colleagues and nurse managers, especially during their troubled times. Participants also reported that promotions were also related to who they know in the organization.

We will be questioned and blamed if the nursing assistant or patient care technician doesn't do their duties right. We have a lot of responsibility with less authority, respect, and support in the system. We are really starving for respect and support. (p 11)

We have a weak nurse who makes various mistakes, and he is still working in the hospital without being re‐evaluated and punished. Everybody knows that he came to the hospital because of his relationship with some authorities. So, it will subconsciously suppress the motivation of other nurses. (p 5)

Many participants also reported being discriminated in the workplace such as an unfair division of duties and work schedules. They voiced that there are no differences between efficient nurses and non‐efficient nurses.

One of our colleagues is off every weekend and holiday, and the rest of the nurses have to work weekends and holidays. Do you think nurses will have any motivation to work? (p 6)

I didn't get any encouragement for my excellent work. Someone who did nothing would get a good reward. Do you think it is fair? (p 1)

Many participants experienced violence from patients, their companions, nursing colleagues and other medical teams. They discussed that violence was in the form of verbal violence, including undermining, ordering, threatening, blaming, overtly criticizing and physical violence such as hitting, slapping, beating and kicking. This violence is continually imposed on nurses in the clinical setting and can overshadow the nurse's self‐actualization in providing ethical nursing care.

One of the patient's companions was very aggressive with me. God knows what would happen if the security did not come to the floor. I was so afraid and confused all night as I didn't know how to care for my patients. (p 3)

#### 3.2.3 Poor working conditions

Poor working conditions were reported to be due to a number of factors related to heavy workload, hostile environment, shortage of nurses and lack of equipment. Participants reported experiencing heavy workload due to inappropriate nurse‐to‐patient ratio and mandatory overtime per month reduced their ability and impart justice in providing care and adherence to professional values.

There is too much patient turnover on our floor, patients being admitted while other patients are being discharged or transferred or became critically ill that needs immediate interventions. Imagine how you could manage your time to even talk to your patients? (p 4)

The experience of heavy workload was also related to the shortage of nursing staff working in the clinical area.

Our hospital has a nursing shortage, and we are forced to work overtime. It's like I work in two places, and as a woman, I have a hard time. Sometimes, I get exhausted and have no energy to talk to my patients. (p3)

Some participants experienced working in a hostile environment that is tense and unfriendly that made them difficult to provide quality nursing care.

One of my colleagues quit her job after working for two years and went home because her work environment was not friendly, and her coworkers didn't have a healthy relationship and did not respect their patients. (p 5)

Lack of equipment can negatively have an impact on the quality of nursing care and endanger patients' lives. One of the participants shared her experience as follows:

We had a 12‐month‐old kid in the operating room. The head nurse asked me to transfer the patient to ICU. We didn't have a proper Ambu Matic ventilator for kids in the ICU. I told my head nurse that transferring this patient with this ventilator was risky. She said, "this is the only one we have here. Go and I hope he will be okay. (p 4)

## 4 DISCUSSION

This study showed that Iranian nurses experience clinical challenges such as a lack of skills in delivering primary care based on modern knowledge and skills. Our findings were in line with Azizinejad (2017) study results, who believed that nurses need continuing education to enhance their knowledge, use technology and evidence‐based research to promote new therapies and strategies. A previous study showed that nurses' knowledge and skills in routine examinations were higher than in less frequently used examinations, which showed that less implementation of these skills justifies poor physical examination skills in nurses (Zeid Abadi et al., 2017). In line with our results, Rizany et al. (2018) study showed that having work experience in different nursing settings and dealing with challenging professional situations can affect a nurse's competence in providing quality nursing care. Specialization in clinical nursing skills is necessary to provide quality care compatible with professional values. Many previous studies confirmed our findings and showed that the skills and expertise of novice nurses were low in the clinical field, and they had more errors and unsafe practices, felt less competent, experienced more stress and reported lower job satisfaction (AlMekkawi & El Khalil, 2020; Fowler et al., 2018).

As a nurse's challenges, value conflict has placed the individual at the crossroads of adherence to professional values and devastatingly affected the individual's presence in the professional nursing setting. Dall'Ora et al. (2020) believed value congruence refers to a match between the job requirements and people's principles. Value conflict between professional value and personal value can cause increased emotional exhaustion in nurses, and this process leads to a high rate of depersonalization and burnout in nurses (Dall'Ora et al., 2020). Similar to our study, the findings of Pursio et al. (2021) study showed that nurses' professional sovereignty, independence in decision‐making and the ability to utilize one's competence affect the freedom to make clinical decisions and accountability. This, in turn, can cause nurses to adhere to the professional values and finally improve the quality of nursing care, nursing satisfaction and retention.

In our study, nurses considered clinical suppressors as one of the underlying factors that can make it difficult to adhere to professional values. Physician dominance and physicians' view of the nursing profession as subordinate and implementer of physician orders are evident in this study. Filizli and Önler (2020) believed that physicians shared the notion that they were still the dominant authority in patient care and they were not comfortable working with nurses in the clinical settings and still saw nurses as their subordinates. Recent studies reported that nurses deny physicians' dominant role, and most nurses had a positive attitude toward physician‐nurse collaboration in inpatients' treatment, which is against our results. A study conducted in Iran showed that cultural and geographical contexts dominate the view of physicians and nurses and may also depend on the clinical settings in which the two groups work together. In another study conducted among Chinese nurses, cooperation between nurses and physicians was more influenced by seniority, and nurses with less seniority were more cooperative with physicians (Cheng et al., 2021; Jasemi et al., 2013). Nurses can adhere to professional values and work based on their values if there is close collaboration between physicians and nurses.

Other factors like repression in the professional environment and violence can hinder the flourishing and development of professional values, and it can overshadow the quality of nursing care delivery and even nurses' job satisfaction. Regardless of the type of violence nurses encounter, their dignity and professional reputation are threatened by patients, their families, colleagues, superiors or physicians. These threats are usually in the form of bullying and humiliation, which often consist of unreasonable job requests, abuse in the workplace, devotion to accepting imposed opinions or views and degrading offers, and have consequences such as mental and physical problems and adverse effects on the social and professional lives of nurses (Hassankhani et al., 2018; Najafi et al., 2017). In addition to violence and suppression of nurses' motivation in clinical settings, discrimination and insufficient support for nurses were also keep the nurses from adhering to their professional values in this study. In line with our results, Afzali et al. (2017) study clearly addresses these issues and declares that an important and influential factor in the destructive behaviours of nurses is creating a sense of organizational injustice, that is, unfair division of work and work schedules. Disrespect for nurses from their superiors, neglect of nurses in the medical system, lack of attention to meritocracy and not promoting the nurses despite sufficient skills are influential factors in creating conditions in which they cannot observe justice and professional values in providing nursing care (Afzali et al., 2017; Hosseinabadi‐Farahani et al., 2021; Najafi et al., 2017).

Another underlying factor affecting nurses' adherence to professional values is the challenging work environment where the nurses provide health care. An atmosphere where the nurse suffers mandatory overtime with a heavy workload in an inappropriate work environment is considered a clinical concern. Similar to Bautista et al.'s (2020) study, a heavy workload is known as the most common stressor in nurses, and high workloads lead to low job satisfaction and reduced quality of nursing care; on the contrary, the heavy workload is positively related to the phenomenon of leaving the profession in nurses (Bautista et al., 2020). In many studies, a positive nursing work environment with sufficient resources is identified as a key element in providing quality nursing care. Increasing the number of nursing personnel and maintaining adequate resources could improve the nurses' work environment (Al Sabei et al., 2020; Griffiths et al., 2018; Kirwan et al., 2013; Liu et al., 2018). Nurses believed that work‐related factors such as heavy workload, insufficient time to perform duties and poor teamwork led to physical, mental and emotional problems (Cooper et al., 2021).

### 4.1 Limitation

In our study, we have not benefited from the experiences of senior nursing managers who play a significant role in professional management and policy‐making; their participation in future similar studies may be helpful and highly recommended. The findings of this study are limited to the factors affecting adherence to professional values in Iranian culture, and further deep qualitative studies are needed to investigate the issue in different cultures. Another study's limitation was that every part of the research process might be influenced by the researchers' cognitive tendencies and filtered through them. The researchers tried to uncover the realities without contaminating them, and they tried not to include their thoughts in identifying and operationalizing the research question, collecting and analysing data and reporting the results as much as possible.

## 5 CONCLUSION

Nurses are affected by negative experiences in clinical settings that can overshadow their professional performance and disrupt their adherence to professional values. Not adhering to professional values can lead to poor quality of patient care and endanger patient safety.

### 5.1 The implication for nursing practice

Nursing managers and health system policymakers must pay attention to nurses' challenges, their professional suppressors and poor working condition in clinical settings to help them promote their professional performance in the health system. Improving the working conditions and clinical atmosphere by recruiting a capable workforce and applying psychological and financial support for nurses is essential to increase the quality of nursing care. The clinical environment would also become a field for nurses to flourish. Thus, nursing managers should not neglect the continuous education of nurses to assist them in increasing their clinical skills by holding practical and theoretical workshops. All medical team members can perform joint workshops to improve interprofessional relationship and cooperation. Nursing managers and physicians can also take fundamental steps to reduce nurses' stress by treating them fairly and respectfully and developing and implementing practical guidelines for preventing violence against them. As a result, nurses' adherence to professional values can improve the quality of nursing care and enhance patient safety.

## AUTHOR CONTRIBUTIONS

SK, NP and HH designed the study. HH and SK conducted the interviews. SK, NP and HH analysed the data. SK and NP wrote the manuscript. All authors read and approved the final manuscript and agreed to be accountable for all aspects of the work.

## FUNDING INFORMATION

This work was financially supported by the Research Council of Urmia Medical Science University (Grant # 10585).

## CONFLICT OF INTEREST

The authors declare that they have no competing interests.

## RESEARCH ETHICS COMMITTEE APPROVAL

All experimental protocol for the research was registered and approved by the ethical committee of Urmia University of Medical Sciences, Iran (IR.UMSU.REC.1399.331).

## Supporting information


Appendix S1

## Data Availability

The data that support the findings of this study are available from the corresponding author upon reasonable request.
