# Supplementary material for: “We are really starving for respect and support,” the struggle of Iranian nurses in adhering to professional values: A qualitative study
Source: Nurs Open. 2023 Jan 30;10(5):3406–14. doi: 10.1002/nop2.1595 (PMC10077361; doi:10.1002/nop2.1595)
Supplement: Supplementary file 1 — Appendix S1 [file NOP2-10-3406-s001.pdf]

Supplementary file
Consolidated criteria for reporting qualitative studies (COREQ): 32-item checklist

Please indicate in which section each item has been reported in your manuscript. If you do not feel an item applies to your manuscript, please enter N/A.

For further information about the COREQ guidelines, please see Tong *et al.*, 2017: https://doi.org/10.1093/intqhc/mzm042

No.	Item	Description	Section #
**Domain 1: Research team and reflexivity**			
Personal characteristics			
1.	Interviewer/facilitator	Which author/s conducted the interview or focus group?	Method/Data coll
2.	Credentials	What were the researcher's credentials? *E.g. PhD, MD*	Method/Data coll
3.	Occupation	What was their occupation at the time of the study?	Method/Data coll
4.	Gender	Was the researcher male or female?	Method/Data coll
5.	Experience and training	What experience or training did the researcher have?	Method/Data coll
Relationship with participants			
6.	Relationship established	Was a relationship established prior to study commencement?	Method/Data coll
7.	Participant knowledge of the interviewer	What did the participants know about the researcher? *E.g. Personal goals, reasons for doing the research*	Method/Data colle
8.	Interviewer characteristics	What characteristics were reported about the interviewer/facilitator? *E.g. Bias, assumptions, reasons and interests in the research topic*	Method/Data colle
**Domain 2: Study design**			
Theoretical framework			
9.	Methodological orientation and theory	What methodological orientation was stated to underpin the study? *E.g. grounded theory, discourse analysis, ethnography, phenomenology, content analysis*	Method/Study des
Participant selection			
10.	Sampling	How were participants selected? *E.g. purposive, convenience, consecutive, snowball*	Method/Participan
11.	Method of approach	How were participants approached? *E.g. face-to-face, telephone, mail, email*	Method/Data colle
12.	Sample size	How many participants were in the study?	Method/Participan
13.	Non-participation	How many people refused to participate or dropped out? What were the reasons for this?	N/A
Setting			
14.	Setting of data collection	Where was the data collected? *E.g. home, clinic, workplace*	Method/Data colle
15.	Presence of non-participants	Was anyone else present besides the participants and researchers?	Method/Data colle

16.	Description of sample	What are the important characteristics of the sample? *E.g. demographic data, date*	Result/Demograph
Data collection			
17.	Interview guide	Were questions, prompts, guides provided by the authors? Was it pilot tested?	Method/Data colle
18.	Repeat interviews	Were repeat interviews carried out? If yes, how many?	Method/Data colle
19.	Audio/visual recording	Did the research use audio or visual recording to collect the data?	Method/Data colle
20.	Field notes	Were field notes made during and/or after the interview or focus group?	Method/Data colle
21.	Duration	What was the duration of the interviews or focus group?	Method/Data colle
22.	Data saturation	Was data saturation discussed?	Method/Participan
23.	Transcripts returned	Were transcripts returned to participants for comment and/or correction?	Method/Rigor
**Domain 3: analysis and findings**			
Data analysis			
24.	Number of data coders	How many data coders coded the data?	Method/Data anal
25.	Description of the coding tree	Did authors provide a description of the coding tree?	Method/Data analy
26.	Derivation of themes	Were themes identified in advance or derived from the data?	Method/Data anal
27.	Software	What software, if applicable, was used to manage the data?	Method/Data analy
28.	Participant checking	Did participants provide feedback on the findings?	N/A
Reporting			
29.	Quotations presented	Were participant quotations presented to illustrate the themes / findings? Was each quotation identified? *E.g. Participant number*	Result/Category/P
30.	Data and findings consistent	Was there consistency between the data presented and the findings?	P7-P19
31.	Clarity of major themes	Were major themes clearly presented in the findings?	P7-P15
32.	Clarity of minor themes	Is there a description of diverse cases or discussion of minor themes?	P7-P19

When submitting your manuscript via the online submission form, please upload the completed checklist as a Figure/supplementary file.

If you would like this checklist to be included alongside your article, we ask that you upload the completed checklist to an online repository and include the guideline type, name of the repository, DOI and license in the *Data availability* section of your manuscript.

Developed from: Allison Tong, Peter Sainsbury, Jonathan Craig, Consolidated criteria for reporting qualitative research (COREQ): a 32-item checklist for interviews and focus groups, International Journal for Quality in Health Care, Volume 19, Issue 6, December 2007, Pages 349–357, https://doi.org/10.1093/intqhc/mzm042
